# Analysis of *Rfo*-Mediated Network in Regulating Fertility Restoration in *Brassica oleracea*

**DOI:** 10.3390/ijms252212026

**Published:** 2024-11-08

**Authors:** Miaomiao Xing, Yuanyuan Xu, Yuyu Lu, Jiyong Yan, Aisong Zeng

**Affiliations:** Institute of Vegetable Crops, Jiangsu Academy of Agricultural Sciences, Jiangsu Key Laboratory for Horticultural Crop Genetic Improvement, Nanjing 210014, China; 20200021@jaas.ac.cn (M.X.); 20130024@jaas.ac.cn (Y.X.); 20070045@jaas.ac.cn (Y.L.); yjyqr@163.com (J.Y.)

**Keywords:** *Rfo*, Ogura CMS, transcriptomics, fertility restoration, tapetum, *Brassica oleracea*

## Abstract

Ogura cytoplasmic male sterility (CMS) lines play a crucial role in the utilization of heterosis. However, valuable traits, such as disease resistance genes from Ogura CMS hybrids, are challenging to incorporate for germplasm innovation, particularly in cabbage and broccoli. To date, the *Rfo*-mediated network regulating fertility restoration remains largely unexplored. In this study, we conducted a transcriptomic analysis of broccoli flower buds from Ogura CMS SFB45 and its *Rfo*-transgenic fertility restoration line, pRfo, at different stages of pollen development. Gene Ontology (GO) terms such as “pollen exine formation”, “flavonoid metabolic and biosynthetic processes”, and “pollen wall assembly”, along with Kyoto Encyclopedia of Genes and Genomes (KEGG) pathways including “flavonoid biosynthesis”, “MAPK signaling pathway-plant”, and “ABC transporters”, were significantly enriched. We identified five differentially expressed genes (DEGs) involved in tapetum-mediated callose metabolism, thirty-four DEGs related to tapetum-mediated pollen wall formation, three DEGs regulating tapetum programmed cell death (PCD), five MPKs encoding DEGs, and twelve DEGs associated with oxidative phosphorylation. Additionally, yeast two-hybrid and bimolecular fluorescence complementation (BiFC) assays demonstrated that RFO directly interacts with ORF138 at the protein level. These findings provide valuable insights into the fertility recovery mechanisms regulated by *Rfo* in broccoli and offer important clues for breeders aiming to enhance Ogura CMS hybrids in *Brassica oleracea*.

## 1. Introduction

Heterosis is critical for the development of high-yielding and resilient crop varieties. Heterosis breeding requires two homozygous inbred lines identified on the basis of general and specific combining abilities [1]. To facilitate hybrid seed production, the female parent needs to be self-incompatible or should be converted into a male sterile line. Male sterility is the most effective tool for heterosis utilization, and it is genetically divided into two types: cytoplasmic male sterility (CMS) and genic male sterility (GMS) [2]. The self-incompatibility and GMS systems have defects in the purity of seed production. Ogura CMS, initially identified in Japanese radish, serves as a pivotal tool in hybrid breeding, particularly for *Brassica* species such as cabbage and broccoli [3]. The stable sterility and complete pollen abortion associated with the Ogura CMS line enable breeders to efficiently produce hybrid seeds, thereby avoiding the labor-intensive process of manual emasculation [4]. Research on pollen development in CMS lines has yielded significant insights into the mechanisms underlying male sterility across various crops, with particular attention to the role of the tapetum. During the early stages of anther development, tapetal cells secrete nutrients, proteins, lipids, and enzymes essential for the development of microsporocytes and microspores. In later stages, these cells provide critical nutrients and signaling molecules necessary for pollen wall formation by initiating programmed cell death (PCD) [5]. Consequently, the precise regulation of tapetal PCD is essential for normal pollen development. Specific mitochondrial genes have been implicated in alterations of tapetal function, leading to delayed or premature PCD in tapetal cells and ultimately resulting in pollen abortion. For instance, the mitochondrial chimeric gene *atp6c* confers CMS in maize by inducing a burst of reactive oxygen species (ROS), which precipitates the premature degeneration of tapetal cells and subsequent pollen abortion [6]. Studies have demonstrated that abnormal PCD in tapetal cells occurs during pollen development in the cabbage Ogura CMS line [7,8]. Given the crucial role of the tapetum in pollen development, numerous genes involved in tapetal development and functional regulation have been well characterized in *Arabidopsis* and other crops, including *TAPETAL DEVELOPMENT and FUNCTION1* (*TDF1*), *POLYKETIDE SYNTHASEA/B* (*PKSA/B*), and *TETRAKETIDE alpha-PYRONE REDUCTASE1/2* (*TKPR1/2*) [5,9,10,11,12,13,14,15,16,17].

The mechanism of fertility restoration involves suppressing the expression of CMS-related genes or eliminating their function. PPR (Pentatricopeptide repeat) proteins play a key role in fertility restoration through RNA editing, mRNA stabilization, and transcript cleavage in various crops. Some PPRs inhibit the translation of sterility-inducing CMS-ORFs, such as Rf1a and Rf1b, which can restore fertility through the endonucleolytic cleavage and degradation of dicistronic *atp6*/*orf79* mRNA in CMS-BT type rice, respectively [18]. ZmRF5 recruits the splicing factor RS31A via MORF8 to form a cleaving/restoring complex that facilitates partial cleavage of the 5′ region of *atp6c*, the CMS-associated transcript [19]. It is well known that the mechanism of fertility restoration in Ogura CMS involves a critical interaction between the fertility restorer gene (*Rfo/PPRB*) and the sterility-causing gene *orf138* [20]. Ogura CMS results from highly rearranged mitochondrial DNAs containing *orf138* [21,22]. The expression of *Rfo* in *Brassica napus* with an Ogura CMS background leads to the complete elimination of ORF138 from the tapetum [23]. Further validation revealed that RFO binds within the coding sequence of mRNA *orf138*, acting as a ribosome blocker to inhibit translation elongation [24].

Despite the advantages and widespread application of the Ogura CMS line in breeding, the extensive utilization of desirable genes from Ogura CMS hybrids for germplasm innovation remains challenging. This is particularly pressing given the shortage of disease-resistant and high-quality germplasm resources in cabbage and broccoli, necessitating the recovery of valuable genetic resources to enhance breeding programs. To maintain the valuable genotypes with Ogura CMS background, somatic embryogenesis can be used [25]. However, for the introduction of these valuable genes into other broccoli inbred lines, explant culture is limited. In cabbage and broccoli, researchers have created Ogura CMS fertility-restored lines through interspecific hybridization and backcrossing [26,27]. The development of Ogura CMS restorers in Brassicaceae vegetable crops has also been conducted via direct transformation of *Rfo* or editing of *ORF138* using mitoTALENs [28,29]. However, these strategies face significant obstacles due to technical challenges and the need to eliminate the genetic background of hybrid materials. Transcriptomic analyses provide a comprehensive view of the regulatory networks associated with pollen development in crops. Chen et al. [8] conducted transcriptome analyses using a normal fertile cabbage line, an Ogura CMS line, and a restorer line containing both the sterility gene *orf138* and the restoration gene *Rfo*. They identified differentially expressed genes (DEGs) related to pollen development, oxidative phosphorylation, and energy metabolism. Zang et al. demonstrated that the DEGs involved in indole biosynthesis, flavonoid biosynthesis, and sugar metabolism exhibit strong network linkages with fertility restoration in cotton CMS-D2 [30]. To elucidate the genetic basis of fertility restoration in the CMS line *WNJ01A* in *Brassica napus*, Yang et al. identified DEGs related to lysine degradation pathways, phenylalanine metabolism, and cutin, suberine, and wax biosynthesis, as well as DEGs involved in tapetum, microspore, and pollen wall development [31].

In this study, we performed transcriptome analyses comparing the Ogura CMS line SFB45 (a widely cultivated broccoli variety) with its fertility-restored line pRfo, obtained via the direct transformation of *Rfo*. Additionally, we confirmed that RFO directly interacts with ORF138 at the protein level. Our findings provide valuable insights into the regulatory mechanisms of fertility restoration mediated by *Rfo*, enhancing our understanding of pollen development processes in broccoli. Furthermore, these results offer valuable gene resources that may facilitate breeders in developing germplasms utilizing Ogura CMS hybrids in *Brassica oleracea*.

## 2. Results

### 2.1. Morphology of the Ogura CMS Line and the Rfo-Transgenic Restorer Line

**Figure 1:**
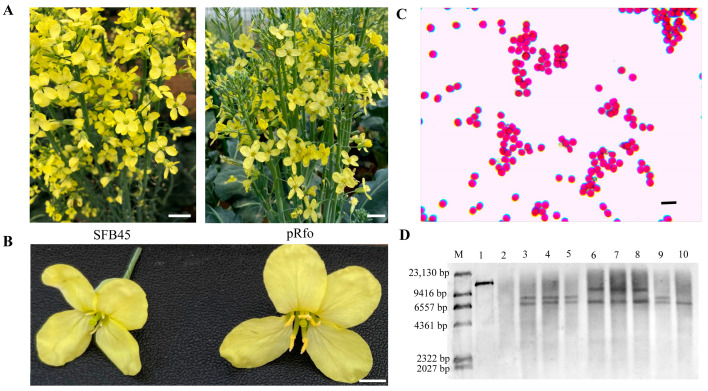
Morphology of the Ogura CMS line SFB45 and the *Rfo*-transgenic restorer line pRfo. (**A**) Comparison of plants. Scale bar = 1 cm. (**B**) Comparison of flowers. Scale bar = 3 mm. (**C**) Pollen viability of pRfo. Scale bar = 50 μm. (**D**) Results of Southern blotting for *Rfo*-transgenic lines. M: marker; Line 1: positive control transgenic carrier vector (*pro*::*Rfo*); Line 2: negative control SFB45; Line 3: pRfo; lines 4–10 represent other *Rfo*-transgenic lines.

The Ogura CMS line SFB45 is a highly resilient and widely cultivated broccoli variety with significant market potential. To restore fertility in SFB45 and develop superior broccoli varieties, we generated its fertility restoration line, pRfo, through the direct transformation of *Rfo*. As shown in Figure 1A,B, the pRfo line produces visible pollen. Alexander staining further demonstrated that the anthers of pRfo contained viable pollen (Figure 1C). Southern blotting confirmed the integration of the *Rfo* gene into the genome of SFB45, and the resulting transgenic lines were classified into three categories based on copy number and insertion positions (Figure 1D).

### 2.2. Overview of RNA-Seq Data Analysis

The transcriptomes of flower buds from the Ogura CMS line SFB45 and its fertility restoration line pRfo, generated through the direct transformation of the *Rfo* gene, were analyzed using RNA-seq. A total of 12 libraries were sequenced, yielding 284,669,194 reads from the SFB45 line and 280,844,976 reads from the pRfo line. All raw reads were deposited in the NCBI database with the accession number PRJNA1169109. The percentage of Q20 quality scores exceeded 97.64%, while the GC content of the clean data ranged from 46.46% to 46.76%. The proportion of mapped reads for each sample ranged from 93.14% to 94.13% when compared to the broccoli reference genome (Table 1).

**Figure 2:**
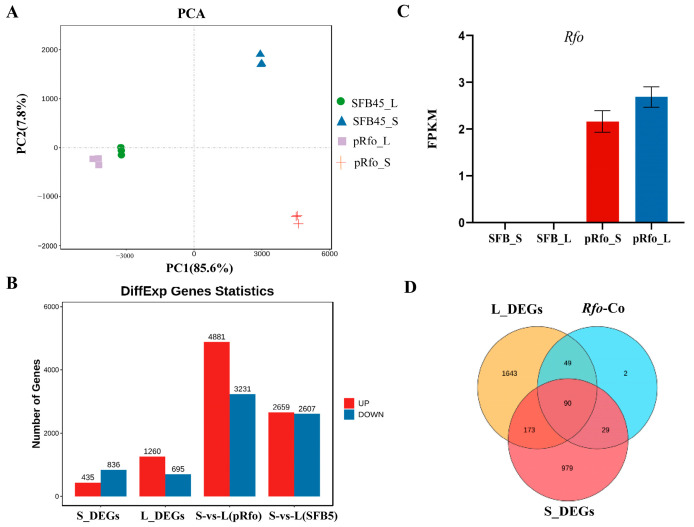
Principal component analysis (PCA) and differentially expressed gene (DEG) statistics. (**A**) PCA clustering based on gene expression. (**B**) Number of DEGs detected in different comparison groups. (**C**) Expression of *Rfo* in different samples. Error bars represent standard deviations. (**D**) Analysis of the shared DEGs in L_DEGs, S_DEGs, and *Rfo* co-expressed genes.

Principal component analysis (PCA) was employed to assess the relationships among the 12 samples. A clear separation between the four groups (SFB45_S, SFB45_L, pRfo_S, and pRfo_L) is evident in Figure 2A, indicating strong reproducibility among these samples. Notably, significant differences were observed between the pRfo_S and SFB45_S samples, suggesting distinct overall transcriptomic profiles.

These results confirm that the quality of the transcriptomic data is reliable and adequate for subsequent analyses.

### 2.3. Screening of Differentially Expressed Genes (DEGs)

DEGs were identified based on the screening threshold (|log2(fold change)| ≥ 1, *p*-value < 0.05) between the pRfo and SFB45 lines. In the small flower bud comparison group (pRfo_S vs. SFB45_S, referred to as S_DEGs), 435 up-regulated and 836 down-regulated DEGs were detected. In the large flower bud comparison group (pRfo_L vs. SFB45_L, referred to as L_DEGs), 1260 up-regulated and 695 down-regulated DEGs were identified (Figure 2B). Among these DEGs, *Rfo* was specifically expressed in the pRfo line as anticipated (Figure 2C).

### 2.4. Analysis of Protein–Protein Interaction (PPI) Networks 

To identify genes that may interact with *Rfo*, we conducted a PPI network analysis using *Rfo* as the hub gene. This analysis revealed 168 co-expressed genes associated with *Rfo* (Figure S1, Table S1). To further explore the molecular mechanisms linked to *Rfo*, we screened DEGs by analyzing shared genes among three gene sets: *Rfo* co-expressed genes, S_DEGs, and L_DEGs. According to the Venn diagram (Figure 2D), 49 *Rfo* co-expressed genes are exclusive to the L_DEGs group, while 29 are exclusive to the S_DEGs group; 90 genes are shared among all three sets. Notably, only two genes identified among the *Rfo* co-expressed genes were not classified as DEGs.

### 2.5. The Enriched Significant Gene Ontology (GO) Pathways

**Figure 3:**
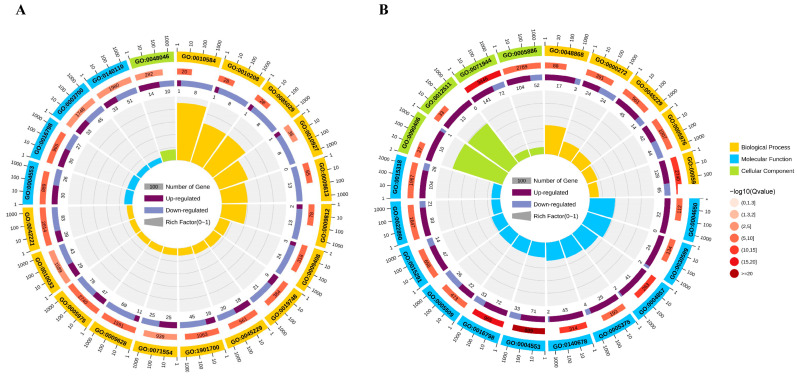
Top 20 of GO enrichment terms of DEGs in SFB45_S vs. pRfo_S. (**A**) and SFB45_L vs. pRfo_L (**B**). Circles from outer to inner sides indicate the following: 1. entry numbers of enriched terms; 2. number of all genes within particular annotated term; 3. number of DEGs within the annotated term (purple block: up-regulated genes; blue block: down-regulated genes); 4. rich factor of the term.

To identify significantly enriched Gene Ontology (GO) pathways associated with *Rfo*-induced DEGs, we analyzed the top 20 enriched pathways for both S_DEGs and L_DEGs. As shown in Figure 3A, the enriched biological processes in S_DEGs predominantly include GO:0010584 (pollen exine formation), GO:0010208 (pollen wall assembly), GO:0085029 (extracellular matrix assembly), GO:0010927 (cellular component assembly involved in morphogenesis), GO:0009813 (flavonoid biosynthetic process), and GO:0009812 (flavonoid metabolic process). In contrast, the primarily enriched biological processes in L_DEGs include GO:0090406 and GO:0048868, which are associated with pollen tube development, as well as GO:0000272 (polysaccharide catabolic process), GO:0045229 (external encapsulating structure organization), and GO:0012511 (monolayer-surrounded lipid storage body) (Figure 3B).

### 2.6. The Enriched Significant Kyoto Encyclopedia of Genes and Genomes (KEGG) Pathways

**Figure 4:**
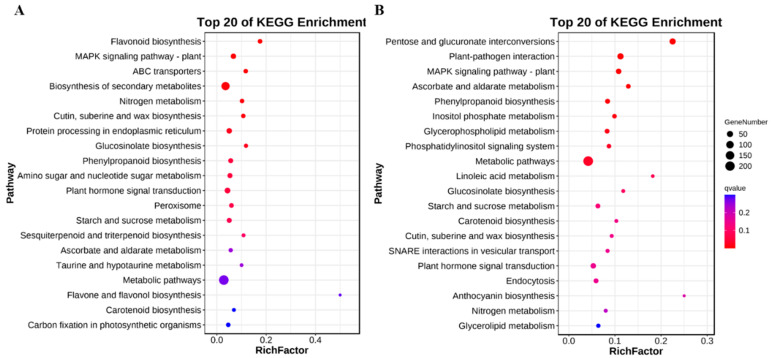
Top 20 KEGG enrichment pathways in SFB45_S vs. pRfo_S (**A**) and SFB45_L vs. pRfo_L (**B**).

To characterize the significantly enriched pathways associated with *Rfo*-induced DEGs, we identified the top 20 enriched Kyoto Encyclopedia of Genes and Genomes (KEGG) pathways in both L_DEGs and S_DEGs (Figure 4). Common pathways between the two groups include “MAPK signaling pathway-plant”, “cutin, suberine and wax biosynthesis”, “phenylpropanoid biosynthesis”, “carotenoid biosynthesis”, and “starch and sucrose metabolism”. Notably, the “MAPK signaling pathway-plant” ranked as the second most enriched pathway in S_DEGs, highlighting the critical role of the MAPK cascade in the development of early flower buds. All these pathways are associated with pollen development and are frequently identified in comparative transcriptomic analyses of male sterile and fertile lines in cabbage and other species, such as wheat [7,32].

In comparison to L_DEGs, the pathways specifically enriched in S_DEGs include “flavonoid biosynthesis”, “ABC transporters”, and “nitrogen metabolism” (Figure 4A). Conversely, the unique significant KEGG pathways for L_DEGs primarily comprise “pentose and glucuronate interconversions”, “plant-pathogen interaction”, “inositol phosphate metabolism”, “glycerophospholipid metabolism”, and “linoleic acid metabolism” (Figure 4B), indicating their involvement in late pollen development.

For the *Rfo* co-expressed genes, KEGG analysis revealed that DEGs are predominantly associated with pathways such as “flavonoid biosynthesis”, “endocytosis”, “protein processing in the endoplasmic reticulum”, and “spliceosome” in addition to those related to pollen wall development (Figure S2). The precise removal of introns from pre-mRNAs is essential for functional mRNA biosynthesis and requires a complex spliceosome [33]. The identified pathways of “spliceosome” and “protein processing in the endoplasmic reticulum” suggest that changes in mRNA and protein biogenesis occur between pRfo and SFB45.

### 2.7. Differentially Expressed Genes (DEGs) Associated with the Tapetum

**Figure 5:**
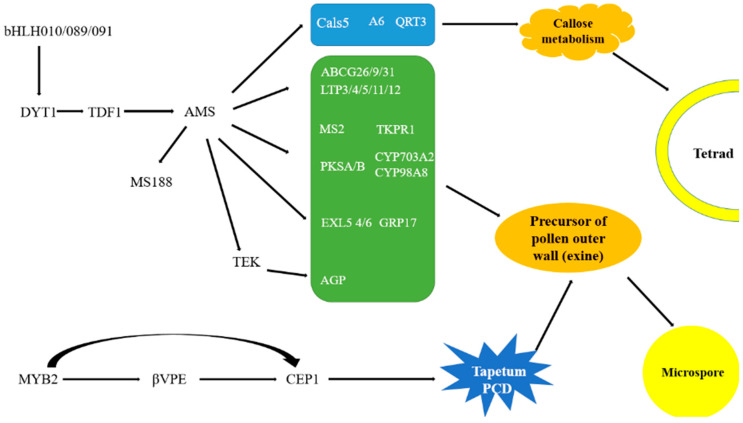
Key genes associated with tapetum development and function that are involved in pollen development. DYT1, DYSFUNCTIONAL TAPETUM1; TDF1, TAPETAL DEVELOPMENT and FUNCTION1; AMS, ABORTED MICROSPORES; TEK, TRANSPOSABLE ELEMENT SILENCING VIA AT-HOOK; Cals5, Callose synthase5; A6, Anther-specific protein6; QRT3, QUARTET3; ABCG, ABC subfamily G; LTP, LIPID TRANSFER PROTEINS; MS2, MALE STERILITY 2; TKPR, TETRAKETIDE alpha-PYRONE REDUCTASE; PKS, POLYKETIDE SYNTHASE; CYP, Cytochrome P450; EXL, EXTRACELLULAR LIPASE; GRP, GLYCINE-RICH PROTEIN; AGP, ARABINOGALACTAN PROTEIN; βVPE, beta VACUOLAR PROCESSING ENZYME; CEP1, CYSTEINE PROTEASE1; PCD, PROGRAMMED CELL DEATH.

**Figure 6:**
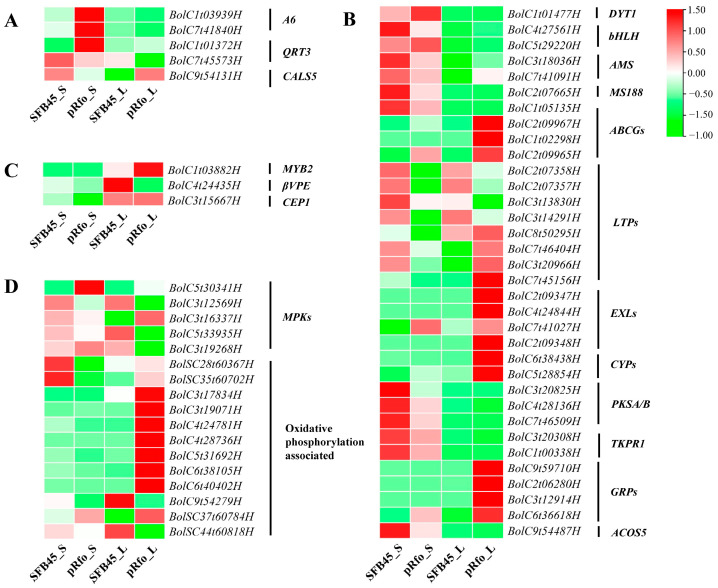
The expression pattern of DEGs involved in tapetum-regulated pollen development. (**A**) Key DEGs involved in tapetum-mediated callose metabolism. (**B**) Key DEGs involved in tapetum-mediated pollen wall formation. (**C**) Key DEGs regulating the programmed cell death of the tapetum. (**D**) Other DEGs related to pollen development. *A6*, *Anther-specific protein6*; *QRT3*, *QUARTET3*; *Cals5*, *Callose synthase5*; *βVPE*, *beta VACUOLAR PROCESSING ENZYME*; *CEP1*, *CYSTEINE PROTEASE1*; *DYT1*, *DYSFUNCTIONAL TAPETUM1*; *AMS*, *ABORTED MICROSPORES*; *ABCG*, *ABC subfamily G*; *LTPs*, *LIPID TRANSFER PROTEINs*; *EXLs*, *EXTRACELLULAR LIPASEs*; *CYPs*, *cytochrome P450*; *PKS*, *POLYKETIDE SYNTHASE*; *TKPR*, *TETRAKETIDE alpha-PYRONE REDUCTASE*; *GRPs*, *GLYCINE-RICH PROTEINs*; *ACOS5*, *acyl-CoA synthetase5*. *MPK*, *Mitogen Activated Protein Kinase*.

To identify key genes associated with tapetum development and function related to pollen development (Figure 5), we searched for DEGs and analyzed their expression patterns (Figure 6, Table S2).

#### 2.7.1. Key DEGs Involved in Tapetum-Mediated Callose Metabolism

The precise timing of callose wall formation and degradation is critical for functional pollen development. The *Callose synthase5* (*CalS5*) gene is responsible for callose synthesis [34]. In this study, we identified a CalS5-encoding gene, *BolC9t54131H*, which is highly up-regulated in the small flower buds of the pRfo line. The tapetum synthesizes and secretes a callase complex to degrade the callose wall surrounding the tetrad microspores [35]. We identified six DEGs associated with callose wall degradation, including two *Anther-specific protein6* (*A6*) genes (*BolC1t03939H*, *BolC7t41840H*), two *QUARTET3* (*QRT3*) genes (*BolC7t45573H*, *BolC1t01372H*), and two *ABORTED MICROSPORES* (*AMS*) genes (*BolC3t18036H*, *BolC7t41091H*) (Figure 6A).

#### 2.7.2. Key DEGs Involved in Tapetum-Mediated Pollen Wall Formation

It is well established that the tapetum synthesizes and secretes materials for pollen wall and pollen coat formation. The development and function of the tapetum are regulated by the well-characterized DYT1-TDF1-AMS-MS188-MS1 module in *Arabidopsis* [36]. Here, we identified one DYSFUNCTIONAL TAPETUM1 (DYT1)-encoding gene, *BolC1t01477H*, which is up-regulated in small buds of pRfo line. Two AMSs encoding DEGs are also identified in this study. One *MS188* (*MYB80*) gene *BolC2t07665H*, exhibited down-regulated expression in pRfo (Figure 6B).

Sporopollenin is the primary component of the sexine layer. Several genes involved in sporopollenin biosynthesis have been identified, including *CYP703A2*, *acyl-CoA synthetase5* (*ACOS5*), *TKPR1*, and *PKSA/B*, and others [37]. In this study, we found that two CYP450-encoding genes, *CYP703A2* (*BolC5t28854H*) and *CYP98A8* (*BolC6t38438H*), are both highly up-regulated in the fertility restore line pRfo. Conversely, the expression of *ACOS5* (*BolC9t54487H*), *PKSA* (*BolC4t28136H*), *PKSB* (*BolC3t20825H*, *BolC7t46509H*), and *TKPR1* (*BolC3t20308H*, *BolC1t00338H*) is significantly reduced in pRfo. Furthermore, *EXTRACELLULAR LIPASE4* (*EXL4*) (*BolC2t09347H*, *BolC4t24844H*), *EXL5* (*BolC7t41027H*) and *EXL6* (*BolC2t09348H*) are all highly up-regulated in pRfo, with *EXL4* and *EXL6* showing no expression in the Ogura CMS line SFB45. Four *GLYCINE-RICH PROTEINs* (*GRPs*) (*BolC9t59710H*, *BolC2t06280H*, *BolC3t12914H*, *BolC6t36618H*) are highly up-regulated in large buds of pRfo, exhibiting a maximum log2 fold change (FC) value of 14.52 (Figure 6B).

Some *ABC subfamily G* (ABCG) members are well characterized in terms of their role in transporting sporopollenin constituents, such as steryl glycosides, across the tapetal plasma membrane to the pollen surface in *Arabidopsis* [38,39]. In this study, *ABCG26* (*BolC1t05135H*) is down-regulated in small buds of pRfo, while *ABCG9* (*BolC1t02298H*) and *ABCG31* (*BolC2t09967H*, *BolC2t09965H*) are up-regulated in large buds of pRfo, particularly *ABCG9*, which is not expressed in SFB45. Lipid transfer proteins (LTPs) represent another well-known subfamily responsible for transporting sporopollenin precursors [40]. Here, we identify several differentially expressed *LTP* genes, including *LTP3* (*BolC3t13830H*, *BolC3t14291H*), *LTP4* (*BolC2t07358H*, *BolC2t07357H*), and *LTP5* (*BolC8t50295H*), all of which are down-regulated in pRfo (Figure 6B).

#### 2.7.3. Key DEGs Regulating the Tapetum PCD

The tapetum PCD is a crucial step in functional pollen development. Studies have shown that the MYB2-βVPE/CEP1 (beta VACUOLAR PROCESSING ENZYME/CYSTEINE PROTEASE1) regulatory module mediates tapetum PCD in *Arabidopsis* [41,42,43,44]. In this study, we identified *MYB2* (*BolC1t03882H*), *βVPE* (*BolC4t24435H*) and *CEP1* (*BolC3t15667H*) as DEGs, with the latter two genes being down-regulated in pRfo (Figure 6C). These results indicate that these three genes are essential for tapetum PCD during pollen development in broccoli.

#### 2.7.4. Other DEGs Related with Pollen Development

The Mitogen-Activated Protein Kinase (MAPK) cascade pathway plays a significant role in tapetal development and pollen fertility [45]. We identified five MAPK-encoding DEGs, including two highly up-regulated *MPK8* (*BolC5t30341H*, *BolC3t16337H*) and three down-regulated *MPK3*, *MPK11,* and *MPK19* genes (*BolC3t12569H, BolC5t33935H, BolC3t19268H*) in pRfo. Twelve oxidative-phosphorylation-associated DEGs were also identified, including *COX1* (*BolSC28t60367H*) and *ndhD* (*BolSC35t60702H*), which are down-regulated in the small flower buds of pRfo, and seven AHA-encoding genes, *ndhB* (*BolSC37t60784H*), and *PPA2* (*BolC9t54279H*), which are up-regulated in the large flower buds of pRfo (Figure 6D).

#### 2.7.5. Validation of Expression of Some DEGs by Quantitative Real-Time PCR (RT-qPCR)

**Figure 7:**
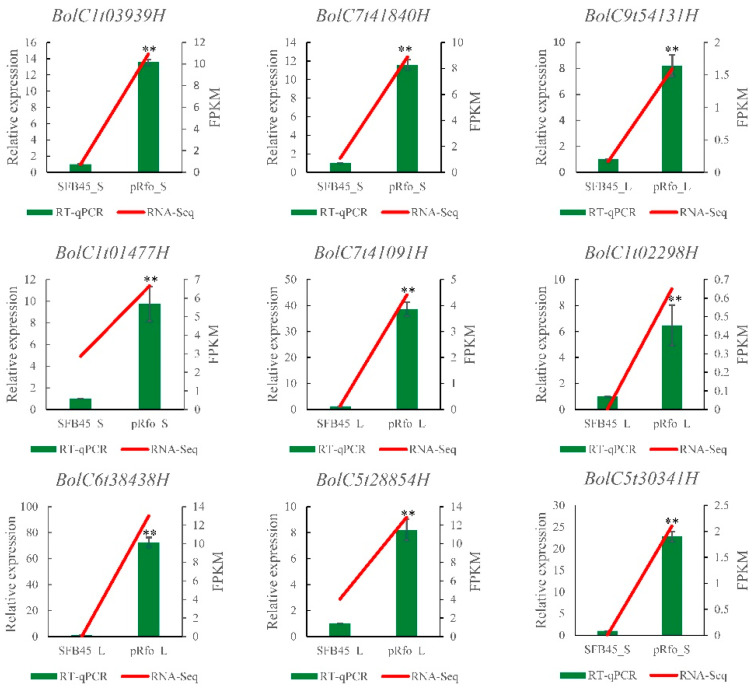
Validation of the expression profiles of the selected DEGs using RT-qPCR. Actin was used as an internal reference gene. Relative gene expression levels were calculated using the 2^−ΔΔCt^ method. Values are means of three independent biological samples and error bars represent standard deviations. Significant differences (*p* ≤ 0.01) based on *t*-test are highlighted by two asterisks. Red represents RNA-seq results based on FPKM values, green represents RT-qPCR results based on relative expression levels.

To validate the expression profiles of the DEGs identified from RNA-seq analysis, nine DEGs significantly up-regulated in fertility restore transgenic line pRfo were randomly selected for RT-qPCR validation. The results showed that expression levels of these genes are significantly up-regulated in either pRfo_S or pRfo_L samples compared to their corresponding sterility line SFB45_S and SFB45_L, respectively, which was consistent with the expression patterns analyzed by RNA-Seq (Figure 7). Our results further supported the high accuracy and reliability of the transcriptomic data.

### 2.8. RFO Directly Interacts with ORF138 at the Protein Level

**Figure 8:**
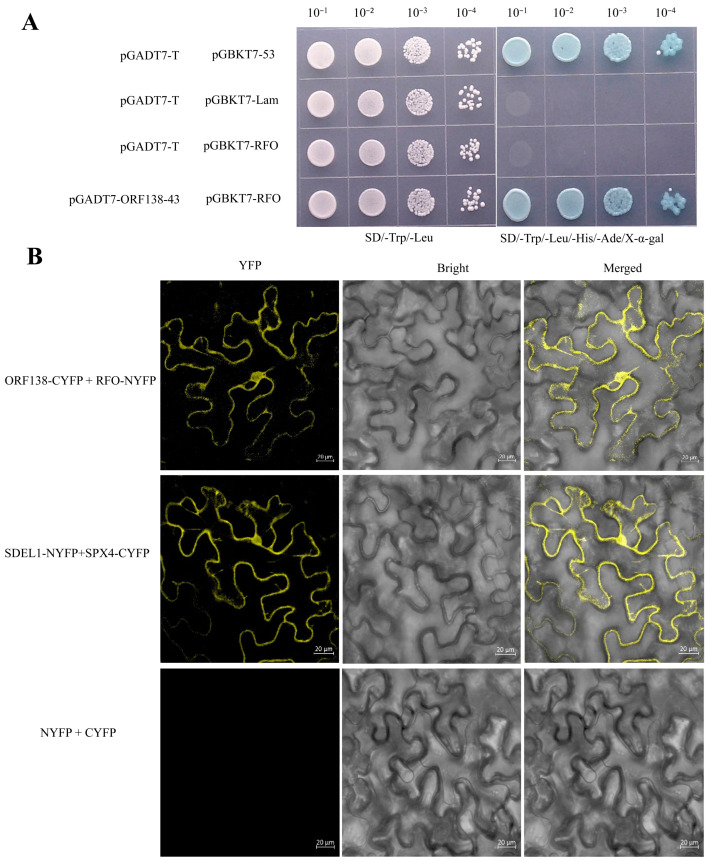
RFO interacts with ORF138 at the protein level in living cells. (**A**) Yeast growth assays on selective media containing the RFO bait and ORF138 prey vectors. (**B**) *N. benthamiana* leaves co-infiltrated with RFO-NYFP and ORF138-CYFP. SDEL1-NYFP and SPX4-CYFP were co-expressed as a positive control. Bar = 20 μm.

Previous studies have shown that the RFO protein can physically interact with the mRNA of *orf138*, effectively inhibiting its function at the translational level [24]. To test whether RFO interacts with ORF138 at the protein level, we cloned the full-length coding sequence (CDS) of *Rfo* and the partail CDS of *orf138* (excluding the N-terminal transmembrane helical domain) to generate pGBKT7-RFO and pGADT7-ORF138-43. A yeast two-hybrid (Y2H) assay was subsequently performed, demonstrating that RFO interacts with the C-terminal of ORF138 (Figure 8A). To further validate this interaction, we cloned the full-length sequences of *Rfo* and *orf138* to create bimolecular fluorescence complementation (BiFC) vectors. The BiFC assay confirmed the binding of RFO to ORF138 (Figure 8B). These results indicate that RFO directly interacts with ORF138 at the protein level and demonstrate that ORF138 is part of the downstream regulatory network mediated by RFO.

## 3. Discussion

Timely tapetum PCD is essential for normal pollen development. Previous studies have demonstrated that in the cabbage Ogura CMS line, tapetal cells compress the microspores at the tetrad stage and subsequently undergo rapid degeneration from the bicellular to the trinucleate microspore stage [7,8]. These findings highlight the abnormal degradation of the tapetum in the Ogura CMS line of *Brassica oleracea*. In this study, we found several KEGG pathways associated with tapetal function to be enriched. Flavonoids, which are synthesized in the tapetum, accumulate in the pollen coat following highly coordinated PCD of the tapetum [46,47]. Furthermore, it has been shown that the restorer gene *Rf1* modulates pollen fertility in cotton CMS-D2 restorer lines via the “flavonoid biosynthesis” pathway [30]. Notably, the “flavonoid biosynthesis” pathway ranks as the most significant pathway in both the *Rfo* co-expressed DEGs and the pRfo_S vs. SFB45_S analysis. The “endocytosis” pathway is the second most significant pathway enriched in the Rfo co-expressed DEGs, corresponding to the biological function of the tapetum, which secretes materials such as nutrients, enzymes, and sporopollenin precursors necessary for pollen development [45]. “ABC transporters” is a specific pathway in pRfo_S vs. SFB45_S analysis. ABCG transporters, a subfamily of ABC transporters, are synthesized in the tapetum to transport sporopollenin precursors from tapetal cells to the pollen wall [36]. These results underscore the indispensable role of the tapetum in the development of early flower buds.

A defective or dysfunctional development of the tapetum often leads to abnormalities in the pollen wall, ultimately resulting in pollen abortion. Mutations in any genes that regulate tapetal development can cause male sterility. In *Arabidopsis*, the dyt1 mutant exhibits abnormal tapetal cells characterized by excessive and/or enlarged vacuoles [48], while the ams mutant demonstrates premature degeneration of both the tapetum and microspores [49]. A similar defect has been observed in the cabbage Ogura CMS line, where tapetal cells exhibit high vacuolation and irregularity during the uninucleate stage [8]. The DEGs encoding DYT1 and AMS identified in this study suggest that these genes play crucial roles in tapetal development in broccoli, reflecting abnormal tapetum development in the Ogura CMS line at the molecular level. The tapetum is known to synthesize and secrete sporopollenin precursors essential for pollen exine and coat formation [50]. Studies have shown that AMS and/or MS188 directly regulate 23 genes involved in pollen wall formation, including *A6*, *QRT3*, *CYP450*, *EXLs*, *ABCG26*, *PKSA/B*, *TKPR1/2*, and *GRPs* [37]. *A6* and *QRT3*, regulated by *AMS*, participate in callose wall degradation [51,52]. *PKSA/B* are co-expressed with *ACOS5*, and together with *TKPR*, they form a conserved biochemical pathway in sporopollenin precursor biosynthesis [53,54,55]. In rice, loss of function of *OsTKPR1* results in delayed tapetum degradation and reduced levels of anther cuticular lipids [56]. In this study, the encoding genes for *PKSA/B*, *ACOS5*, and *TKPR1* are all down-regulated in the small flower buds of pRfo.

Analyzing the expression patterns of genes, especially among certain subfamilies, enables us to characterize the biological functions of each member at specific developmental stages. Notably, we found that the ABCG26-encoding gene is down-regulated in the small buds of pRfo, while ABCG9/31-encoding genes are up-regulated in the large buds of pRfo. This corresponds to their sequential functions: ABCG26 is required for pollen exine formation [38,57], while ABCG9 and ABCG31 are involved in pollen coat deposition [39]. Among the LTP-encoding DEGs, *LTP3/4/5* are down-regulated in pRfo buds, while *LTP11/12* are specifically expressed in large buds of pRfo, suggesting different regulatory roles for these *LTP* genes during pollen development. Similarly, among the *EXLs*, three *EXL4/6* encoding genes show specific expression in large flower buds of pRfo, whereas one *EXL5* gene exhibits up-regulated expression in small flower buds of pRfo. Although EXL4/5/6 are characterized as pollen coat proteins, *EXL4*, which functions with *GRP17* to promote pollen hydration and efficient pollination [58,59,60], differs from the other two, whose functions remain uncharacterized. Based on their expression patterns, *EXL5* is likely a structural component, while *EXL4/6* may serve as functional constituents for viable pollen. The expression patterns of these genes suggest diverse and sequential roles in different stages of pollen development in broccoli. Our results indicate that abnormal changes occur in early flower buds (<3.5 mm) of the Ogura CMS line.

The enrichment of the MAPK signaling pathway in both GO and KEGG analyses highlights its significant role in broccoli pollen development. In wheat CMS lines, excess ROS leads to delayed initiation of tapetal PCD and ultimately results in pollen abortion [61]. It has been reported that *MPK8* negatively regulates ROS accumulation in *Arabidopsis* [62]. In our study, three MPK8-encoding genes are highly up-regulated in the pRfo line, suggesting that *MPK8* plays a role in maintaining ROS homeostasis during pollen development in broccoli. Other MAPK subfamilies, including *MPK3*, *MPK11*, and *MPK19*, are all down-regulated in the pRfo line; the roles of these members in broccoli pollen development remain unclear.

Fertility restoration occurs at various molecular levels, including transcriptional, post-transcriptional, translational, and post-translational levels, etc. [63]. An exemplary case of transcriptional restoration has been demonstrated by the recently identified fertility restorer gene *Rfs*, which encodes a PPR protein consisting of 15 PPR motifs that binds to and cleaves the *orf138* mRNA of Type H [64]. The post-transcriptional restoration mechanism is exemplified by *Rfo*, which binds to the CDS of the CMS-causing *orf138* mRNA, inhibiting its translation into a toxic protein [24]. However, no protein-level interaction between RFO and ORF138 has been reported prior to this study. Here, in our study, we validated that RFO can directly interact with ORF138 at the protein level through BiFC and Y2H assays. Our results reveal another new molecular mechanism of fertility recovery mediated by RFO at the protein level, in addition to the previously reported translation inhibition mechanism that RFO binds within the CDS of *orf138* to inhibit its elongation. It indicates that a dual molecular mechanism of fertility recovery is activated by RFO at both the translational and post-translational levels, ensuring the complete elimination of ORF138 and restoration of fertility. So far, no such dual mechanism has been found. In the RT98-type CMS of rice, multiple genes (excluding *PPR762*) are involved in the restoration process for full fertility recovery [65]. This suggests that the plant fertility recovery mechanism is complex and finely regulated.

## 4. Materials and Methods

### 4.1. Plant Material and Sample Collection

In this study, the Ogura CMS line SFB45, a widely cultivated broccoli variety Naihanyouxiu (trade name in China), released by Sakata Seed Corporation (Japan), was selected for the transformation of the fertility gene *Rfo* to generate its fertility-restored line pRfo. For transformation of *Rfo*, a fragment 4064 bp in length containing the promoter region (2000 bp) and the *Rfo* gene-coding region (2064 bp) was inserted into a modified pBWA(V)BS vector to generate *pRfo::Rfo*. The construct was introduced into *Agrobacterium tumefaciens* strain GV3101 via chemical transformation following the instructions and then was transformed into SFB45, as described by Liu et al. [66]. Transgenic seedlings were selected according to their Basta resistance and PCR amplification using primers for Bar. The two lines SFB45 and pRfo were grown at Liuhe scientific research base in Nanjing. All plants were grown in open fields from September to December and then transplanted to greenhouses. After vernalization under cold conditions (0–10 °C), they flowered in April of the following year.

Flower buds from the SFB45 and pRfo lines were categorized into four groups: buds measuring less than 3.5 mm in length (designated as SFB45_S and pRfo_S) and buds measuring 3.5 mm or greater (designated as SFB45_L and pRfo_L). All samples were promptly frozen in liquid nitrogen and stored at −80 °C with three biological replications for each group.

### 4.2. Southern Blot Analysis

To confirm the transgenic insertion of *Rfo* into the SFB45 genome and to assess the copy number, a Southern blot assay was conducted, as described in a previous study [67]. DNA was extracted from the flower buds of both the pRfo and SFB45 lines and digested with *SacI*. The transgenic carrier vector (*prfo::Rfo*) was included as a positive control, digested with *BamHI*.

### 4.3. Observation of Pollen Viability

The pollen viability of the pRfo line was assessed using an Alexander staining kit (Beijing Baikai Biology Technology Co., Ltd., Beijing, China) as described by Chen et al. [8]. Five newly opened flowers at full bloom were collected, and the pollen was spread onto a microscope slide, followed by the addition of eight drops of the staining solution. After 30 min, the stained pollen was examined under an OLYMPUS BX41 microscope (Tokyo, Japan). The assay was conducted with three technical replicates.

### 4.4. RNA Extraction and Sequencing

Total RNA was extracted from 12 samples (SFB45_S1, SFB45_S2, SFB45_S3, pRfo_S1, pRfo_S2, pRfo_S3, SFB45_L1, SFB45_L2, SFB45_L3, pRfo_L1, pRfo_L2, pRfo_L3) using the RNAprep Pure Plant Kit (Tiangen Biotechnology, Beijing, China), following the manufacturer’s instructions. After evaluating the purity and integrity of the extracted RNA, mRNA was enriched using mRNA capture beads and subsequently fragmented using high temperatures. The fragmented mRNAs were then utilized to construct cDNA libraries. Hieff NGS^®^ DNA Selection Beads were employed for purification to select the adapter-ligated target fragments. PCR library amplification was performed, followed by sequencing on the Illumina NovaSeq X Plus platform [68].

### 4.5. Sequencing Data Analysis and Identification of DEGs

To obtain high-quality clean reads for further analysis, raw reads were filtered using fastp (version 0.18.0) [69]. The filter parameters were as follows: (1) removal of containing adapters and ploy-N; (2) exclusion of low-quality reads with more than 50% of bases having a Q-value ≤ 20; (3) elimination of reads mapped to the ribosomal RNA database. The paired-end clean reads were then aligned to the broccoli reference genome “Braol HDEM V 1.0” (http://www.brassicadb.cn/#/ (accessed on 1 November 2024)) using HISAT2 (version 2.1.0) [70]. FPKM (fragment per kilobase of transcript per million mapped reads) values in SFB45_S, pRfo_S, pRfo_L and SFB45_L samples were calculated to quantify the gene expression by RSEM software (V 1.2.19) [71]. Based on gene expression data, PCA was conducted using R package gmodels (http://www.r-project.org/ (accessed on 1 November 2024)). DEGs were identified in different comparing groups (pRfo_S vs. SFB45_S, pRfo_L vs. SFB45_L, pRfo_S vs. pRfo_L and SFB45_S vs. SFB45_L) based on the FPKM values of transcripts in different samples using DESeq2 [72] software with the criteria of |log2(foldchange)| ≥ 1 and *p* < 0.05.

### 4.6. Protein–Protein Interaction

*Rfo* co-expressed genes were identified using the STRING database to analysis the PPI network using String v10 [73], designating Rfo as the node and interactions as lines in the network. A protein interaction network with confidence > 0.7 was built and was visualized using Cytoscape (v3.7.1) [74] to illustrate biological interactions.

### 4.7. Functional Annotations of DEGs 

DEGs were mapped to GO terms in the Gene Ontology database (http://www.geneontology.org/ (accessed on 1 November 2024)) and KEGG pathways (http://www.genome.jp/kegg/ (accessed on 1 November 2024)). The GOseq R package and KOBAS 2.0 were used to identify significantly enriched terms or pathways with *p*-value (FDR) < 0.05 [75].

### 4.8. Yeast Two-Hybrid Assay

The yeast two-hybrid assay was conducted as described by Wang et al. [76]. The full-length CDS of *Rfo* and the partail CDS of *ORF138* (excluding the first 129 bp) were separately cloned into pGBKT7 and pGADT7 vectors using Golden Gate cloning. The two constructs were then co-transformed into the yeast strain AH109. Prior to the experiments, auto-activation of the pGBKT7-RFO and pGADT7-ORF138-43 constructs was tested. The transformed colonies were plated onto triple dropout (TDO: -Trp, -Leu, -His) and quadruple dropout (QDO: -Trp, -Leu, -His, -Ade) media supplemented with X-α-Gal for visual growth assessment. Positive (pGBKT7-53 and pGADT7-T) and negative (pGBKT7-Lam and pGADT7-T) controls were performed in parallel. The plates were incubated at 30 °C for 5–7 days for final observation.

### 4.9. BiFC Assay

The full-length CDSs of *Rfo* and *orf138* lacking a stop codon were subcloned into the pCAMBIA1300-35S-NY173 and pCAMBIA1300-35S-YC155 vectors to generate RFO-NYFP and ORF138-CYFP, respectively. Both recombinant plasmids were introduced into Agrobacterium tumefaciens strain EHA105 and were transiently co-expressed at a 1:1 ratio in the leaves of 4-week-old tobacco seedlings through agro-infiltration. Positive (SDEL1-NYFP and SPX4-CYFP) and negative (pCAMBIA1300-NYFP and pCAMBIA1300-CYP) controls were processed similarly [77]. After 3 days of growth, YFP fluorescence was monitored using a laser confocal microscope (NIKON-C2, Tokyo, Japan).

### 4.10. RT-qPCR Analysis

The same RNA samples that were used for RNA-Seq were used for RT-qPCR. The first-strand cDNA synthesis was conducted using the PrimeScript^TM^ RT reagent kit with gDNA eraser (TaKaRa, Beijing, China). RT-qPCR was performed using the AceQ Qpcr SYBR Green Master Mix (Vazyme, Nanjing, China) on a Bio-Rad CFX96TM Real-Time System (Bio-Rad, Hercules, CA, USA). The quantification of the RT-qPCR data was achieved using the 2^−ΔΔCT^ method [78]. Actin (GenBank accession number XM_013731369.1) was used as an internal control to normalize the expression level. The relative expression levels of the selected genes in Rfo_S or in Rfo_L were obtained by comparing with that in SFB45_S or in SFB45_L, respectively. Values are means of three independent biological samples of SFB45_S, pRfo_S, pRfo_L and SFB45_L and error bars represent standard deviations. Statistical significance between two samples was determined by *t*-test using Graphpad Prism 8.0 software, with ** *p* < 0.01 indicating high significance. The primer sequences can be found in Table S3.

## 5. Conclusions

In this study, we conducted a transcriptomic analysis of broccoli flower buds from Ogura CMS and its *Rfo*-transgenic fertility restore line. Significantly enriched GO terms and KEGG pathways included those related to pollen exine formation, pollen wall assembly, flavonoid metabolic and biosynthetic, MAPK signaling, and ABC transporters. We identified key DEGs, particularly those involved in tapetum development and metabolite pathways, which play crucial roles in pollen development. Furthermore, Y2H and BiFC assays demonstrated that RFO can directly interact with ORF138 at the protein level. Our findings will facilitate further exploration of the *Rfo*-regulated fertility recovery mechanism in broccoli, as well as the identification of potential gene targets for the enhancement of the germplasm through Ogura CMS hybrids in *Brassica oleracea*.

## Figures and Tables

**Table 1:** Summary and evaluation of transcriptomic data.

Sample ID	Total Reads	Clean Reads	GC Content (%)	Q20	Mapped Reads (%)
SFB45_L1	43,562,004	43,275,336	46.63%	97.88%	93.58%
SFB45_L2	52,851,156	52,452,396	46.60%	97.70%	93.55%
SFB45_L3	51,435,408	51,037,542	46.60%	97.82%	93.70%
SFB45_S1	51,794,632	51,411,684	46.49%	98.16%	94.13%
SFB45_S2	46,112,684	45,680,624	46.48%	97.64%	93.14%
SFB45_S3	41,202,802	40,811,612	46.46%	97.77%	93.41%
pRfo_L1	54,675,592	54,353,582	46.47%	97.97%	94.13%
pRfo_L2	43,224,430	42,933,156	46.55%	97.91%	93.73%
pRfo_L3	47,846,634	47,502,090	46.51%	97.93%	93.78%
pRfo_S1	48,812,596	48,375,164	46.75%	97.95%	93.49%
pRfo_S2	40,532,022	40,148,548	46.76%	97.87%	93.44%
pRfo_S3	47,956,782	47,532,436	46.75%	97.78%	93.61%

## Data Availability

The original contributions presented in this study are included in the article/Supplementary Materials; further inquiries can be directed to the corresponding author.

## Supplementary Materials

The following supporting information can be downloaded at https://www.mdpi.com/article/10.3390/ijms252212026/s1, Figure S1. *Rfo* regulatory network based on protein–protein interaction analysis. Figure S2. KEGG pathways of *Rfo*-coexpressed genes. Table S1. *Rfo*-coexpressed genes. Table S2. Key DEGs involved in pollen development in this study. Table S3. Primers used for RT-qPCR.
