# Supplementary figures and images for: Analysis of Rfo-Mediated Network in Regulating Fertility Restoration in Brassica oleracea
Source: Int J Mol Sci. 2024 Nov 8;25(22):12026. doi: 10.3390/ijms252212026 (PMC11593589; doi:10.3390/ijms252212026)

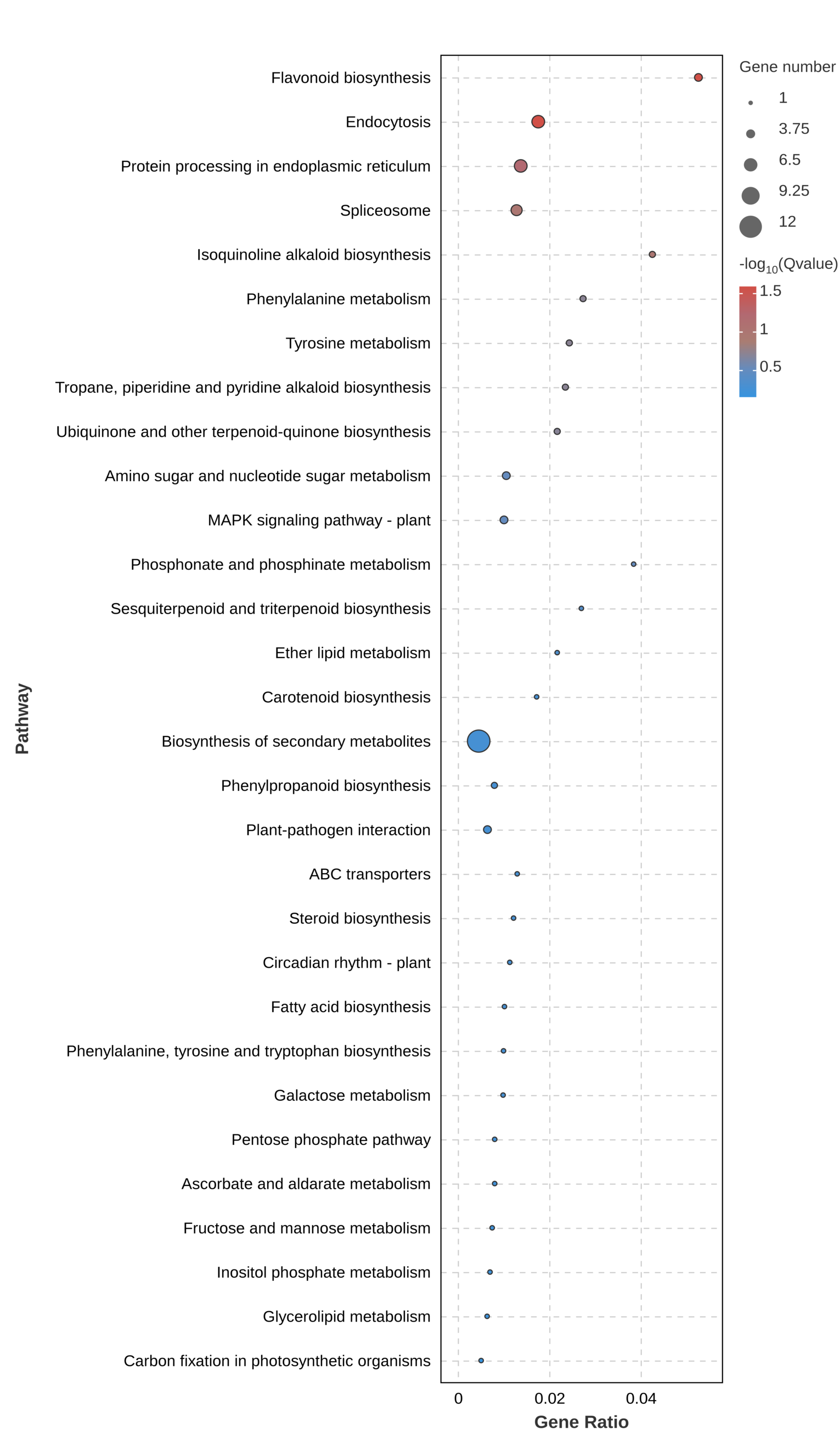

Supplement: Supplementary file 1 [file ijms-25-12026-s001.zip › supplement files/Figure S1. KEGG pathways of Rfo-coexpressed genes.tiff]
